# Integration of scRNA-Seq and Bulk RNA-Seq Reveals Molecular Characterization of the Immune Microenvironment in Acute Pancreatitis

**DOI:** 10.3390/biom13010078

**Published:** 2022-12-30

**Authors:** Zhen Fang, Jie Li, Feng Cao, Fei Li

**Affiliations:** Department of General Surgery, Xuanwu Hospital, Capital Medical University, Beijing 100053, China

**Keywords:** acute pancreatitis, single-cell RNA-sequencing, bulk RNA-sequencing, immune microenvironment, molecular characterization

## Abstract

Acute pancreatitis (AP) is an acute inflammatory disease of the exocrine pancreas. The pathogenesis of AP is still unclear, and there is currently no specific treatment. A variety of immune cells infiltrate in AP, which may play an important role in the progression of the disease. In this study, for the first time, scRNA-Seq and Bulk RNA-Seq data were used to show the characteristics of immune cell infiltration in AP, and to explore the specific molecular markers of different cell types. The present study also investigated cell-to-cell communication networks using the CellChat package, and AP-specific gene signatures (Clic1, Sat1, Serpina3n, Atf3, Lcn2, Osmr, Ccl9, Hspb1, Anxa2, Krt8, Cd44, Cd9, Hsp90aa1, Tmsb10, Hmox1, Fxyd5, Plin2, Pnp) were identified through integrative analysis of multiple sequencing datasets. We also defined disease-specific associated genes in different cell types, revealing dynamic changes through cell trajectory and pseudo-time analysis using the Monocle2 package. The results showed that macrophages were significantly increased in acute pancreatitis, and the number of interactions and interaction weight/strength of the macrophages in AP were significantly higher than those in the controls. The activities of various signaling pathways were abnormally regulated such as apoptosis, oxidative stress, lysosome, autophagy, ferroptosis, and inflammatory responses signaling pathways. In conclusion, this study comprehensively depicted the immune microenvironment of AP, explored the interaction network between different cell types, and defined AP-specific gene signatures, providing many new directions for basic research in AP.

## 1. Introduction

Acute pancreatitis (AP) is characterized by severe inflammation and acinar cell death, with an incidence rate of 13–45/100,000 [1,2,3]. About 20% of AP patients eventually develop severe organ failure, with a mortality rate between 15% and 35% [4]. Current treatments are mainly early fluid resuscitation, pain control, nutritional support, and anti-infection. However, because most treatments are based on symptoms, specific and effective treatments are still needed. The etiology of AP is complex and diverse, involving immune cell infiltration and the release of various inflammatory factors. Therefore, understanding the characteristics of immune cell infiltration and discovering specific marker genes are of great value for the treatment of acute pancreatitis in the future.

Single-cell RNA sequencing (scRNA-Seq) uses next-generation sequencing technology to define the global gene expression profile of a single cell, which helps to dissect the distribution characteristics of different types of cell populations [5]. Bulk RNA-Seq can reveal tissue-wide transcriptional expression profiles, but it masks differences between individual cells. Emerging scRNA-Seq reveals the expression profiles of individual cells, complementing the shortcomings of bulk RNA-Seq. The two are integrated for analysis, and new discoveries may be made. There are very few studies of single-cell sequencing for pancreatitis. Several studies have used single-cell sequencing technology to characterize single-cell expression profiles in chronic pancreatitis [6,7,8]. However, it has not been reported in acute pancreatitis.

In this study, we revealed the distribution and molecular characteristics of immune microenvironment populations in AP, and validated the expression of marker genes in pancreatic tissue. At the same time, we also analyzed the interaction between different cell subsets, confirming that monocytes–macrophages play an important role in AP. The AP-related disease-specific gene signature was revealed by integrating scRNA-Seq and Bulk RNA-Seq, revealing the key target genes of acute pancreatitis.

## 2. Materials and Methods

### 2.1. Datasets, Animal, and Reagents

We downloaded the GSE188819, GSE65146, and GSE109227 datasets from the GEO database (https://www.ncbi.nlm.nih.gov/geo/, accessed on 1 September 2022). We used the single-cell sequencing data of four samples in the GSE188819 dataset, two samples from the control group, and two samples from the cerulein group. In addition, the RNA-sequencing data of eight samples from the GSE65146 dataset and 11 samples from the GSE109227 dataset were used for analysis. All of these data are available in the Supplementary Materials. C57BL/6 mice (SPF, 6 weeks old, 20 ± 2 g) were purchased from the WeiTongLiHua experimental animal technical company (Beijing, China). The mice were fed with standard feed and water, the ambient temperature was about 25 °C, and the ambient humidity was about 50%. Newly arrived mice had free access to food and water for at least 3 days. Fasting started 12 h before the experiment. The animal experiment was approved by the Ethics Committee of Xuanwu Hospital of Capital Medical University (XW20211223-1). Cerulein (HY-A0190, MCE, NJ, USA). Antibody: Clic1 (1:100; #53424, CST, Danvers, MA, USA), Atf3 (1:500; ab305293, Abcam, Boston, MA, USA).

### 2.2. Animals Model

To elicit acute pancreatitis, mice were intraperitoneally injected with cerulein (HY-A0190, MCE) every 1 h at a dosage of 50 μg/kg body weight per injection, seven times a day for two consecutive days. Cerulein was diluted with saline for administration (*n* = 5). Control mice were injected with the same volume of normal saline at the same time (*n* = 5). Mice were kept fasted but allowed water during the experiment. One hour after the last injection, mice were euthanized by spinal dislocation and collected for pancreatic tissue.

### 2.3. scRNA-Seq Data Preprocessing and Integration

Different cells were isolated by digesting pancreatic tissue with 1 mg/mL collagenase P, 2 U/mL dispase II, 0.1 mg/mL soybean trypsin inhibitor, and 0.1 mg/mL DNase I in HBSS with Ca^2+^/Mg^2+^. Tissue dissociation was performed using a gentle MACS™ Octo Dissociator at 37 °C for 40 min. Tissue was digested with 0.05% trypsin-EDTA for an additional 5 min at 37 °C and red blood cells were removed with red blood cell lysis buffer. We followed the steps in the manufacturer’s protocol (Chromium Single Cell 3’ GEM, Library & Gel Bead Kit v3, PN-1000075) to load cells onto 10× Chromium Single Cell Controller Chip B (10× Genomics). This was the method used by the authors in the source article to isolate cells [9]. The GSE188819 dataset was downloaded from the GEO database. Data quality control and preprocessing were performed using the Seurat package (v4.0.2, Rahul Satijaa, Oxford, UK, https://satijalab.org/seurat/, accessed on 1 September 2022). Genes were removed from the red blood cells: Hba1, Hba2, Hbb, Hbd, Hbe1, Hbg1, Hbg2, Hbm, Hbq1, and Hbz. NormalizeData(), FindVariableFeatures(), and ScaleData() were applied to normalize the scRNA-Seq data. The filtered scRNA-Seq datasets of CER and the control sample were integrated using the Seurat anchor-based integration method [10], based on the expression of the 2000 most variable features of each sample.

### 2.4. Dimensionality Reduction, Clustering, Visualization, and Cell Type Recognition

Seurat v4 in R 4.0.3 was used to perform dimensionality reduction, clustering, and visualization for the scRNA-Seq data. Principal component analysis (PCA) was used for aa reduction in the dimensionality of the integrated data. On the basis of the first 10 principal components (PCs), the integrated dataset was further reduced to two-dimensional (2D) space and visualized by T-distributed stochastic neighbor embedding (tSNE). FindNeighbors(), FindClusters(), and RunTSNE() were used to perform these analyses with a resolution of 0.1–0.5. According to the canonical patterns of marker genes, the 29,071 cells were annotated as 15 cell types including macrophages, fibroblasts, acinar cells, endothelials, monocytes, neutrophils, progenitor cells, ductal cells, dendritic cells (DC-cell), stellate cells, α-cells, β-cells, T cell, B cell, and granulocytes. The main reference databases of cell marker genes for cell annotation came from CellMarker (http://xteam.xbio.top/CellMarker/index.jsp, accessed on 1 September 2022) and CellMarker2.0 (http://bio-bigdata.hrbmu.edu.cn/CellMarker/index.html, accessed on 1 September 2022).

### 2.5. AUCell Gene Set Enrichment Analysis

We used the AUCell package to calculate the AUCell score to signal the signature genes for each individual cell within these clusters and display interactive t-SNE maps of the resulting scores.

### 2.6. Analysis of Single-Cell Trajectories

The single-cell pseudotime trajectories were generated with the Monocle2 package in R4.0.3. The newCellDataSet(), estimateSizeFactors(), and estimateDispersions() were used to perform these analyses. The detectGenes() was used to filter low quality cells with “min_expr = 0.1”.

### 2.7. Cell–Cell Crosstalk Network Analysis

We used CellChat to investigate the molecular interaction networks between different cell types [11]. CellChat is a tool to quantitatively infer and analyze cell-to-cell communication networks from scRNA-Seq data. Ligand–receptor pairs with a *p*-value of less than 0.05 determined by CellChat were considered to be significant interacting molecules between different cell subpopulations.

### 2.8. Bulk RNA-Seq Data Analysis

The GSE65146 and GSE109227 datasets were downloaded from the GEO database. A Venn diagram was made to depict the overlap between differentially expressed genes from the GSE188819, GSE65146, and GSE109227 datasets.

### 2.9. Differentially Expressed Genes (DEGs) Analysis

Genes specific to each cluster or group were identified using the “FindAllMarkers” function, and adjusted *p*-values were calculated using the Wilcoxon rank-sum test. Volcano plots were used to show the fold changes and log-adjusted *p*-values for DEGs. tSNE visualization was used to depict the expression of the overlap gene signature.

### 2.10. Gene Ontology (GO) and Kyoto Encyclopedia of Genes and Genomes (KEGG) Analysis

We used the R package “clusterProfiler” to perform Gene Ontology (GO) and Kyoto Encyclopedia of Genes and Genomes (KEGG) analyses. “enrichGO”, “enrichKEGG”, and genome-wide annotation packages “org.Mm.eg.db” were needed. A *p*-value of < 0.05 was considered significant enrichment.

### 2.11. Gene Sets Variation Analysis (GSVA)

Gene set variation analysis (GSVA) was implemented via the ‘gsva’ Bioconductor package. The analysis based on a non-parametric unsupervised approach, which transformed a classic gene matrix (gene-by-sample) into a gene set by the sample matrix resulted in an enrichment score for each sample and pathway. The GSVA matrix was then clustered and displayed as a heat map using the “Pheatmap” package.

### 2.12. Histology Staining

Mice pancreas were excised and fixed with 4% paraformaldehyde for 48 h. After paraffin embedding, the pancreas was cut into 5-μm-thick sections. The prepared sections were stained with hematoxylin-eosin (HE) and immunohistochemical (IHC) and photographed under a microscope. Simply put, for immunohistochemical (IHC) analysis, pancreatic tissue slides were deparaffinized and rehydrated through an alcohol series followed by antigen retrieval with sodium citrate buffer. Immunohistochemical staining for anti-Clic1 (1:100; #53424, CST, USA) and Atf3 (1:500; ab305293, Abcam, USA) were conducted at 4 °C overnight, and then the secondary antibody was incubated for one hour at room temperature. Diaminobenzidine (DAB) was used for color counter-staining. Images were taken with an Olympus (Tyoko, Japan).

## 3. Results

### 3.1. Single-Cell Transcriptional Profiling of Pancreatic Cells

**Figure 1:**
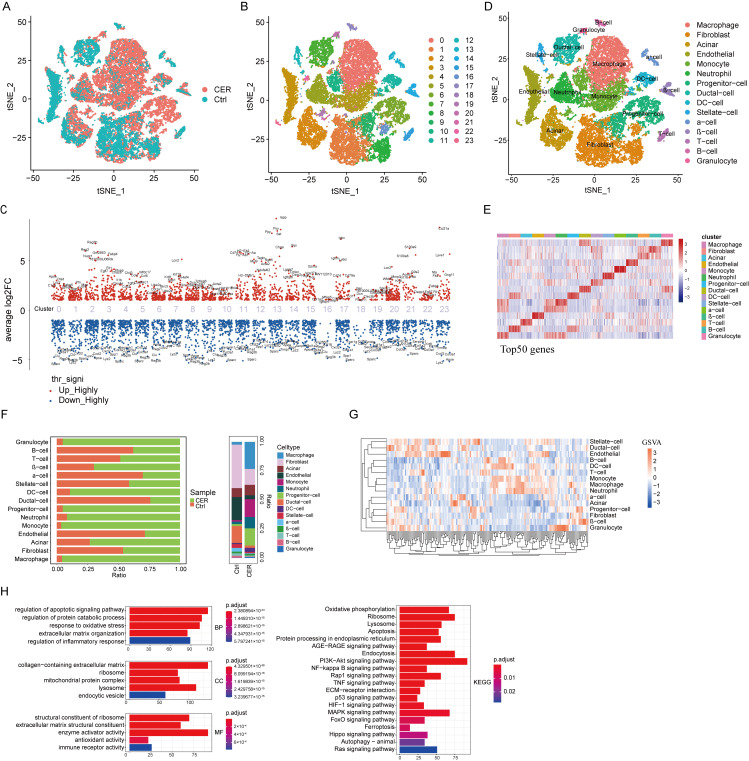
Overview of the single-cell transcriptional profiling of pancreatic cells. (**A**) tSNE visualization of the CER and control samples. (**B**) tSNE visualization of 24 cell clusters. (**C**) Marker genes of the 24 cell clusters. (**D**) tSNE visualization of macrophages, fibroblasts, acinar cells, endothelials, monocytes, neutrophils, progenitor cells, ductal cells, DC-cells, stellate cells, α-cells, β-cells, T cells, B cells, and granulocytes. (**E**) Heatmap visualization of the top 50 genes of 15 cell types. (**F**) The distribution of cells derived from different cell types and different sample origins. (**G**) GSVA analysis in 15 cell types. (**H**) GO and KEGG enrichment analysis for CER and the control.

A total of 29,071 cells were analyzed from the C57BL/6 mouse pancreas samples, of which 8668 cells were from the control mouse pancreases and 20,403 cells were from the AP mouse pancreases. We employed tSNE for dimensionality reduction, clustering, and visualization of the datasets (Figure 1A. Twenty-four major cellular clusters were identified by scRNA-sequencing, represented using tSNE analysis (Figure 1B). The marker genes of each cluster were analyzed by the “FindAllMarker” package (Figure 1C and Figure S1). According to the canonical patterns of marker genes, the 29,071 cells were annotated as 15 cell types including macrophages, fibroblasts, acinar cells, endothelials, monocytes, neutrophils, progenitor cells, ductal cells, DC-cells, stellate cells, α-cells, β-cells, T cells, B cells, and granulocytes (Figure 1D, Table S1). Heatmaps were drawn based on differential genes for different cell types (Figure 1E, Table S2). The distribution of cells derived from different cell types and different sample origins is shown in Figure 1F. The proportion of macrophages, monocytes, and neutrophils in the CER group was higher than their proportion in the control group (Figure 1F) This may indicate that they play an important role in acute pancreatitis [12,13,14]. Pathway analyses were conducted by gene set enrichment analysis (GSEA). The results showed that the activity of various signaling pathways in the immune cells was significantly increased such as apoptosis, oxidative-phosphorylation, lysosome, and Toll-like -receptor signaling pathways (Figure 1G and Figure S2). In addition, we performed GO and KEGG enrichment analysis and found that signaling pathways such as apoptosis, oxidative stress, lysosome, autophagy, ferroptosis, and inflammatory responses were significantly enriched (Figure 1H). Taken together, CER exhibited a higher enrichment of the regulation of inflammatory response, apoptotic, and oxidative stress signatures than the control.

### 3.2. Single-Cell RNA-Seq Analysis Reveals Characterization of Acinar Cells

**Figure 2:**
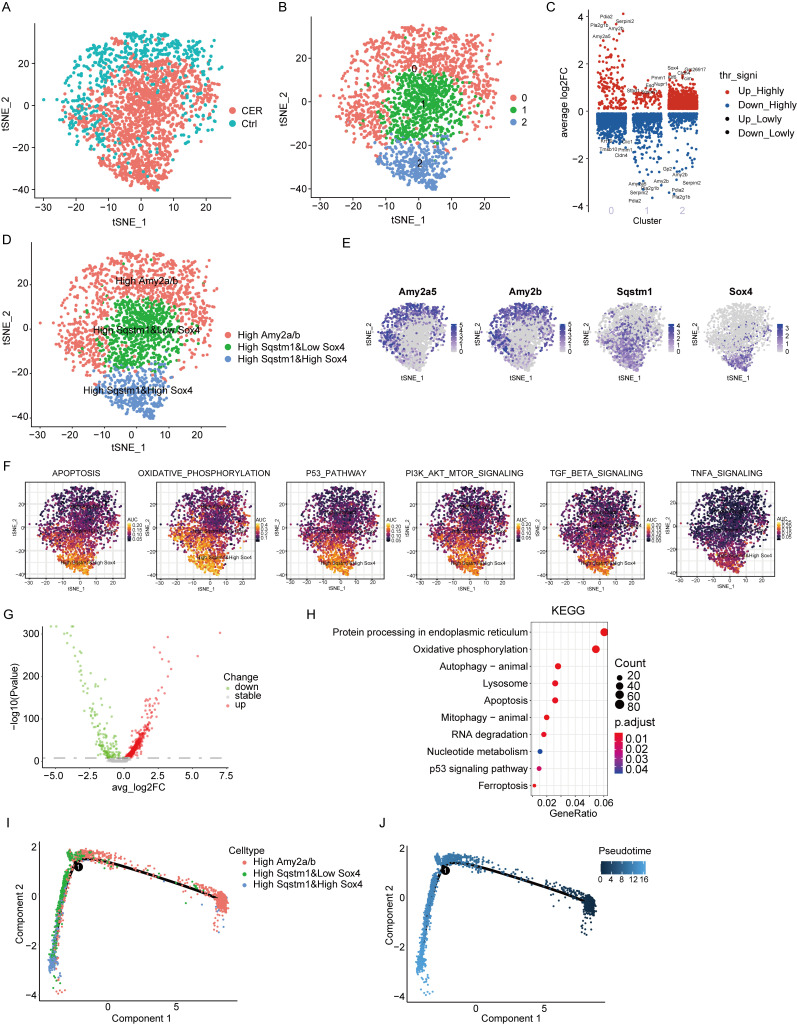
Single-cell RNA-Seq analysis reveals biological characteristics of acinar cells. (**A**) tSNE visualization of acinar cells from CER and the control samples. (**B**) tSNE visualization of three cell subclusters. (**C**) Marker genes of three cell subclusters. (**D**) tSNE visualization of Amy2a/b^high^, Sqstm1^high^ and Sox4^low^ and Sqstm1^high^ and Sox4^high^ acinar cells. (**E**) The expression of marker genes in tSNE visualization. (**F**) The AUCell package was used to calculate the AUCell score. (**G**) The volcano map shows the differential gene profiling expression. (**H**) The bubble chart of KEGG pathway enrichment analysis. (**I**,**J**) Trajectory analysis of Amy2a/b^high^, Sqstm1^high^ and Sox4^low^ and Sqstm1^high^ and Sox4^high^ acinar cells using Monocle2.

Acinar cells have a crucial role in the development of AP [1]. Acinar cell populations were extracted for subcluster analysis. CER and the control samples were demonstrated using the tSNE method (Figure 2A). Three main cell clusters were identified, represented using tSNE analysis (Figure 2B). The marker genes of each subcluster were analyzed by the “FindAllMarker” package (Figure 2C). The distribution of acinar cells derived from different cell clusters and different sample origins is shown in Figure S3. Three cell types were identified based on marker genes: Amy2a/b^high^, Sqstm1^high^, and Sox4^low^, and Sqstm1^high^ and Sox4^high^ acinar cells (Figure 2D,E). The Amy2a/b^high^ subcluster was mainly composed of normal acinar cells. The AUCell package was used to calculate the AUCell score for signaling pathway signature genes for each individual cell within these subclusters. We found that Sqstm1^high^ and Sox4^low^ and Sqstm1^high^ and Sox4^high^ subclusters had higher AUCell scores in apoptosis, oxidative-phosphorylation, p53, PI3K-AKT-mTOR, TGF-β, and TNF-α signatures than the Amy2a/b^high^ subcluster, and was more significant in the Sqstm1^high^ and Sox4^high^ subcluster (Figure 2F). KEGG enrichment analysis was performed based on differential genes derived from CER and the control sample of acinar cells. The results showed that differential genes were significantly enriched in oxidative-phosphorylation, autophagy, lysosome, apoptosis, P53, and ferroptosis signaling pathways (Figure 2G,H). Trajectory analysis of Amy2a/b^high^, Sqstm1^high^ and Sox4^low^, and Sqstm1^high^ and Sox4^high^ acinar cells using Monocle2. Sqstm1^high^ and Sox4^high^ acinar cells were found at later developmental time points (Figure 2I,J). The above evidence suggests that Sqstm1 and SOX4 may play an important role in the progression of acute pancreatitis. Studies have found that autophagy is involved in the evolution of acute pancreatitis, and Sqstm1/p62 is an important regulator of the autophagy process [1,15]. In AP, the role of SOX4 has not been reported.

### 3.3. Single-Cell RNA-Seq Analysis Reveals Characterization of Monocytes

**Figure 3:**
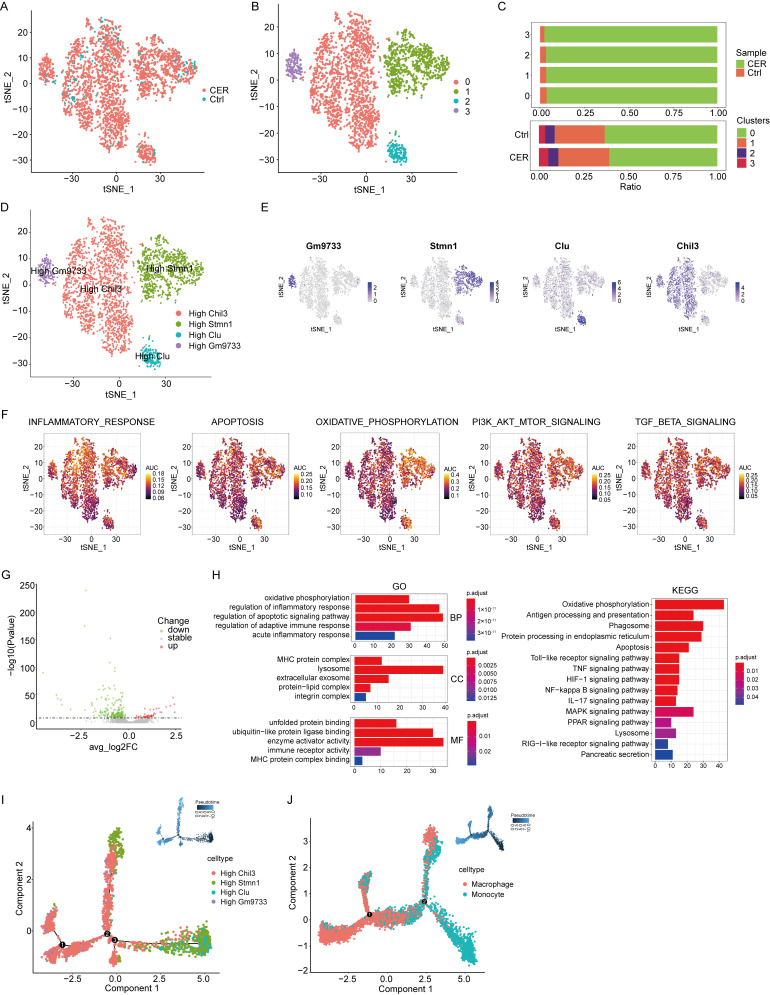
Single-cell RNA-seq analysis reveals biological characterization of monocytes. (**A**) tSNE visualization of monocytes from CER and Control samples. (**B**) tSNE visualization of 4 cell subclusters. (**C**) The distribution of cells derived from different cell clusters and different sample origins. (**D**) tSNE visualization of Chil3^high^, Stmn1^high^, Clu^high^, and Gm9733^high^ monocytes. (**E**) The expression of marker genes in tSNE visualization. (**F**) AUCell package was used to calculate the AUCell score. (**G**) The volcano map show that the differential gene profiling expression. (**H**) The bar graph of GO and KEGG pathway enrichment analysis. (**I**) Trajectory analysis of Chil3^high^, Stmn1^high^, Clu^high^, and Gm9733^high^ monocytes using Monocle2. (**J**) Trajectory analysis of monocytes and macrophages using Monocle2.

Monocytes populations were extracted for subclusters analysis. CER and Control samples were demonstrated using the tSNE method. (Figure 3A) 4 main cell clusters were identified, represented using tSNE analysis. (Figure 3B) The distribution of monocytes derived from different cell types and different sample origins were showed in Figure 3C. 4 cell types were identified base on marker genes: Chil3^high^, Stmn1^high^, Clu^high^, and Gm9733^high^ monocytes. (Figure 3D,E) A study reported that the expression of clusterin (Clu) in the serum of AP patients was significantly increased [16]. Chil3 and Stmn1 have been reported in pancreatic cancer but not in AP [17,18]. There are high AUCell score in inflammatory response, apoptosis, oxidative phosphorylation, PI3K-AKT-mTOR, TGF-β signatures, and more significant in Stmn1^high^ subcluster. (Figure 3F) The differential genes were subjected to GO and KEGG enrichment analysis, and the results showed that they were significantly enriched in oxidative phosphorylation, inflammatory response, phagosome, apoptosis, and NF-kappa B (NF-κB) signaling pathways. (Figure 3G,H) Trajectory analysis of Chil3^high^, Stmn1^high^, Clu^high^, and Gm9733^high^ monocytes using Monocle2. Chil3^high^ monocytes were found at later developmental time points. (Figure 3I) Integrating data from monocytes and macrophages, using Monocle2 for cell trajectory analysis, showed monocyte-to-macrophage differentiation (Figure 3J).

### 3.4. Single-Cell RNA-Seq Analysis Reveals Characterization of Macrophages

**Figure 4:**
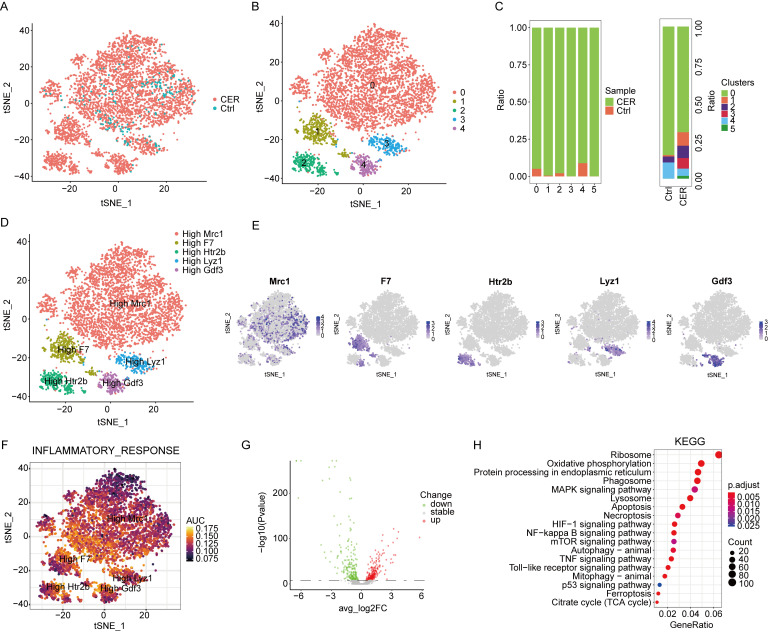
Single-cell RNA-Seq analysis reveals biological characterization of macrophages. (**A**) tSNE visualization of macrophages from CER and the control samples. (**B**) tSNE visualization of five cell subclusters. (**C**) The distribution of cells derived from different cell clusters and different sample origins. (**D**) tSNE visualization of Mrc1^high^, F7^high^, Htr2b^high^, Lyz1^high^, and Gdf3^high^ macrophages. (**E**) The expression of marker genes in tSNE visualization. (**F**) The AUCell package was used to calculate the AUCell score. (**G**) The volcano map shows the differential gene profiling expression. (**H**) The bubble chart of the KEGG pathway enrichment analysis.

In the immune microenvironment of AP, macrophages account for the largest proportion and play a crucial role in the progression of AP. Macrophage populations were extracted for subcluster analysis. CER and the control samples were demonstrated using the tSNE method (Figure 4A). Five main cell clusters were identified, represented using tSNE analysis (Figure 4B). The distribution of macrophages derived from different cell clusters and different sample origins is shown in Figure 4C. In the CER sample, the proportion of macrophages was significantly higher than that in the control sample. The marker genes of each cluster were analyzed by the “FindAllMarker” package. Five cell types were identified based on marker genes: Mrc1^high^, F7^high^, Htr2b^high^, Lyz1^high^, and Gdf3^high^ macrophages (Figure 4D,E). One study suggests that the anti-inflammatory cells of MRC1+ macrophages may be dysfunctional in a mouse model of neuropathic pain, and that returning these cells to a normal state may help with treatment [19]. However, MRC1+ macrophages have not been reported in the study of acute pancreatitis. Inflammatory response signature genes were found to have a high AUCell score by using the AUCell package (Figure 4F). KEGG enrichment analysis was performed based on differential genes derived from CER and the control macrophages, and it was found that they were significantly enriched in ribosome, oxidative phosphorylation, phagosome, MAPK, apoptosis, autophagy, NF-κB, and p53 signaling pathways (Figure 4G,H). In conclusion, consistent with the current findings, inflammation-related signaling pathways are activated in AP including apoptosis, autophagy, NF-κB signaling pathways, and so on.

### 3.5. Single-Cell RNA-Seq Analysis Reveals Characterization of Neutrophil

**Figure 5:**
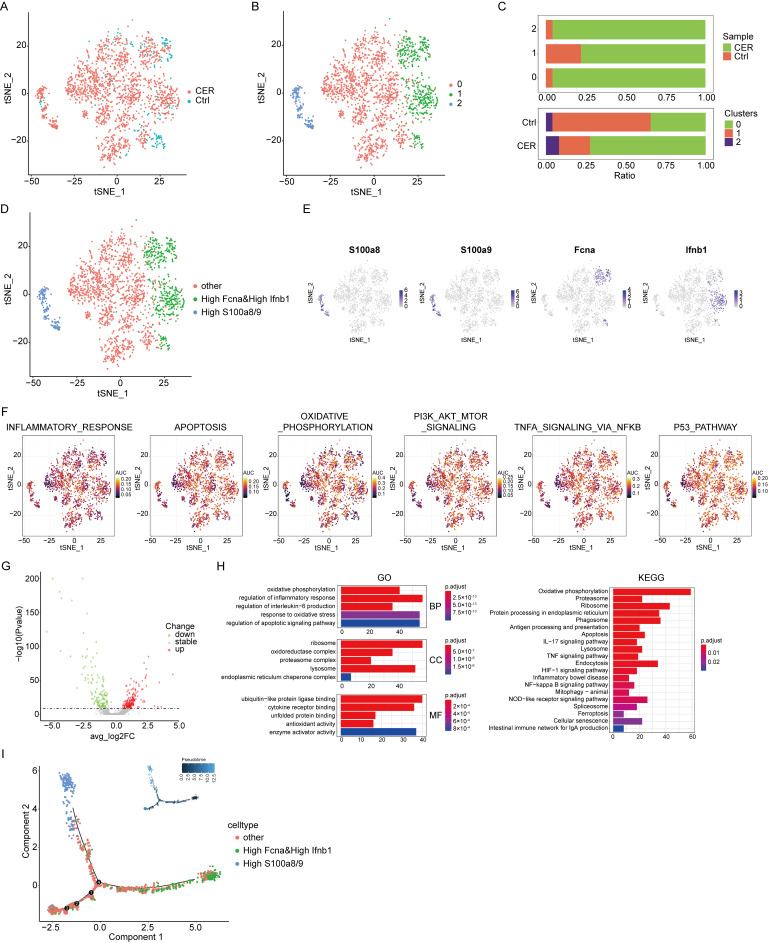
Single-cell RNA-Seq analysis reveals biological characterization of neutrophil. (**A**) tSNE visualization of neutrophils from CER and the control samples. (**B**) tSNE visualization of three cell subclusters. (**C**) The distribution of cells derived from different cell clusters and different sample origins. (**D**) tSNE visualization of Fcna and Ifnb1^high^, S100a8/9^high^, and “other” neutrophils. (**E**) The expression of marker genes in tSNE visualization. (**F**) The AUCell package was used to calculate the AUCell score. (**G**) The volcano map shows the differential gene profiling expression. (**H**) The bar graph of the KEGG pathway enrichment analysis. (**I**) Trajectory analysis of Fcna and Ifnb1^high^, S100a8/9^high^, and “other” neutrophils using Monocle2.

There are three types of granulocytes: neutrophils, eosinophils, and basophils. We all know that neutrophils play an important role in acute inflammation diseases. Therefore, we grouped neutrophils individually and performed an in-depth analysis. Neutrophil populations were extracted for subcluster analysis. CER and the control samples were demonstrated using the tSNE method (Figure 5A). Three main cell clusters were identified, represented using tSNE analysis (Figure 5B). The distribution of neutrophils derived from different cell clusters and different sample origins is shown in Figure 5C. Three cell types were identified based on marker genes: Fcna^high^ and Ifnb1^high^, S100a8/9^high^, and “other” neutrophils (Figure 5D,E). Oxidative phosphorylation and the TNF-α signature genes were found to have a high AUCell score by using the AUCell package. (Figure 5F) The differential genes were subjected to GO and KEGG enrichment analysis, and the results showed that they were significantly enriched in oxidative phosphorylation, inflammatory response, apoptosis, PI3K-AKT, and the TNF signaling pathways (Figure 5G,H). Trajectory analysis of Fcna^high^ and Ifnb1^high^, S100a8/9^high^, and “other” neutrophils was conducted using Monocle2. S100a8/9^high^ neutrophils were found at later developmental time points (Figure 5I). In conclusion, a variety of inflammation-related signaling pathways in neutrophils were activated during AP and promoted the progression of AP. S100a8/9 may play an important role in the biological function of neutrophils.

### 3.6. Single-Cell RNA-Seq Analysis Reveals Characterization of Fibroblast

**Figure 6:**
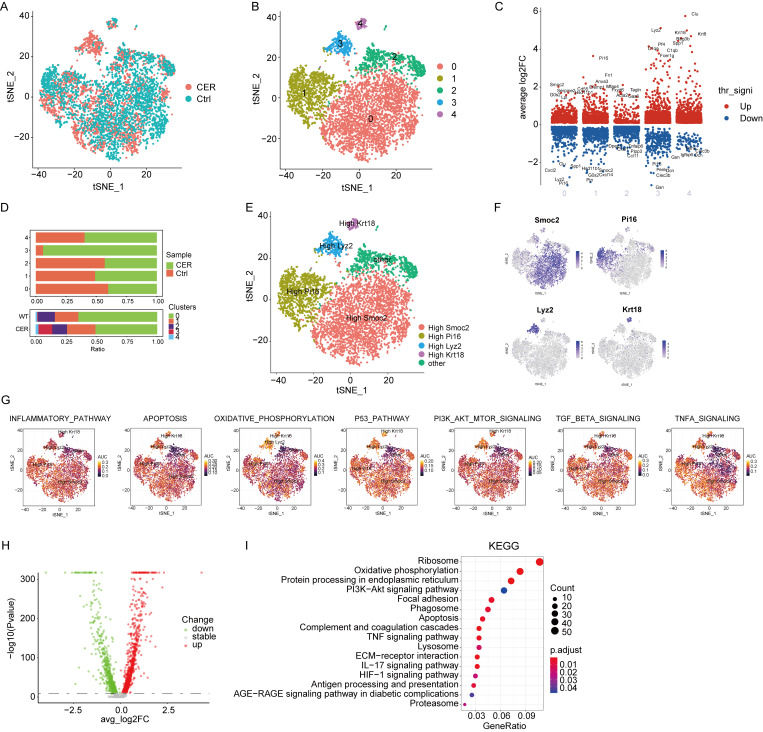
Single-cell RNA-Seq analysis reveals biological characterization of fibroblasts. (**A**) tSNE visualization of fibroblasts from CER and the control samples. (**B**) tSNE visualization of five cell subclusters. (**C**) Marker genes of five cell subclusters. (**D**) The distribution of cells derived from different cell clusters and different sample origins. (**E**) tSNE visualization of Smoc2^high^, Pi16^high^, Lyz2^high^, Krt18^high^, and other fibroblasts. (**F**) The expression of marker genes in tSNE visualization. (**G**) The AUCell package was used to calculate the AUCell score. (**H**) The volcano map shows the differential gene profiling expression. (**I**) The bubble chart of the KEGG pathway enrichment analysis.

Fibroblast populations were extracted for subcluster analysis. CER and the control samples were demonstrated using the tSNE method (Figure 6A). Five main cell clusters were identified, represented using tSNE analysis (Figure 6B). The marker genes of each subcluster were analyzed by the “FindAllMarker” package (Figure 6C). In the CER sample, the proportion of the “3” subcluster was significantly higher than that in the control sample (Figure 6D). Five cell types were identified based on marker genes: Smoc2^high^, Pi16^high^, Lyz2^high^, Krt18^high^, and “other” fibroblasts (Figure 6E,F). The study found that the expression of Krt18 was significantly increased in mild acute pancreatitis [20]. Smoc2 may play an important role in chronic pancreatitis [21]. There were high AUCell score in inflammatory response, apoptosis, oxidative phosphorylation, P53, PI3K-AKT-mTOR, TGF-β, and TNF-α signatures (Figure 6G). The differential genes were subjected to GO and KEGG enrichment analysis, and the results showed that they were significantly enriched in ribosome, oxidative phosphorylation, PI3K-AKT, apoptosis, and the TNF signaling pathways (Figure 6H,I).

### 3.7. Single-Cell Transcriptional Analysis Reveals the Cell–Cell Crosstalk Network

**Figure 7:**
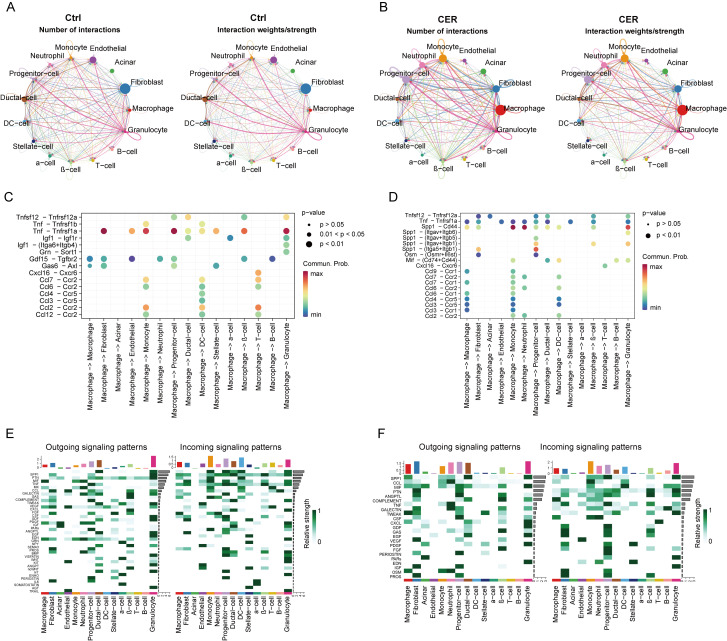
Single-cell transcriptional analysis reveals the cell–cell crosstalk network. (**A**,**B**) Analysis of the number of interactions and interaction strength among different cell types in the control sample and CER sample. (**B**) Analysis of the number of interactions and interaction strength among different cell types in the CER sample. (**C**,**D**) Identification of signaling by comparing the communication probabilities mediated by ligand–receptor pairs from macrophages to other cell types in the control sample and CER sample. (**E**,**F**) Overview of the outgoing signaling and incoming signaling in CER and the control sample.

To clarify the underlying intercellular communications and cell state transitions in the pancreas, we analyzed the intercellular communication networks from the scRNA-Seq data using the CellChat package (Figure 7A,B). We detected many significant ligand–receptor pairs among the 15 cell types. In the CER sample, the number of interaction and interaction weight/strength of the macrophages was significantly higher than that of the control sample. This result suggests that macrophages play an important role in acute pancreatitis. Furthermore, identifying altered ligand–receptor pairs from macrophages to other cell types by comparing their communication probabilities between the control and CER sample (Figure 7C,D). We explored important ligand–receptor pairs sent from the macrophages to other cell types in the CER sample compared to the control sample. We found that TNF signaling such as Tnfsf12-Tnfrsf12a was increased from the macrophages to acinar cells in the CER sample compared to the control sample. CCL signaling such as ccl9-ccr1, ccl7-ccr1, ccl6-ccr1, and ccl3-ccr1 were increased from macrophages to monocytes in the CER sample. We also overviewed the outgoing signaling and incoming signaling in CER and the control sample. The main outgoing signals in macrophages were MIF, PDGF, and EDN signaling in the control sample, and SPP1, CCL, MIF, TWEAK, CXCL, and OSM signaling in the CER sample (Figure 7E,F). The above results indicate that macrophage-mediated TNF, SPP1, and CCL signaling play an important role in AP.

### 3.8. Integrated Analysis in scRNA-Seq and Bulk RNA-Seq Data Reveals Gene Signature

**Figure 8:**
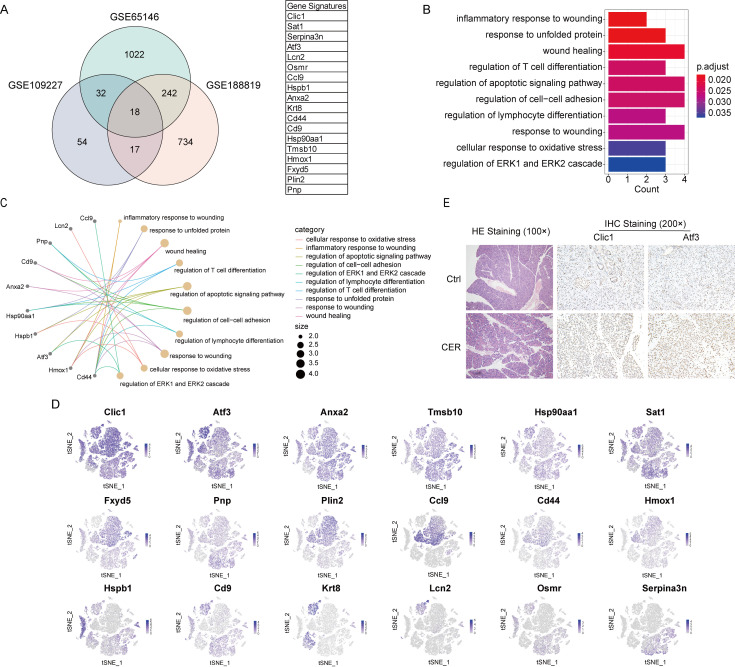
Integrated analysis in scRNA-Seq and bulk RNA-Seq data reveals the gene signature. (**A**) A Venn diagram was produced to depict the overlap genes (Data from the GSE188819, GSE65146, and GSE109227 datasets). (**B**,**C**) GO enrichment analysis and visualization. (**D**) The expression of gene signature in tSNE visualization. (**E**) HE and IHC staining.

A Venn diagram was produced to depict the overlap between differentially expressed genes from the GSE188819, GSE65146, and GSE109227 datasets. AP-specific gene signatures (Clic1, Sat1, Serpina3n, Atf3, Lcn2, Osmr, Ccl9, Hspb1, Anxa2, Krt8, Cd44, Cd9, Hsp90aa1, Tmsb10, Hmox1, Fxyd5, Plin2, Pnp) were found (Figure 8A). These genes were significantly enriched (p. adjust < 0.05) in the genes of 10 GO pathways such as inflammatory response to wounding, response to unfolded protein, wounding healing, regulation of the apoptotic signaling pathway, and cellular response to oxidative stress (Figure 8B,C). We also depict the expression of gene signature in tSNE visualization. Clic1, Atf3, Anxa2, Tmsb10, and Sat1 were expressed in various cell subtypes, while other genes were expressed in only a few cell types (Figure 8D). Immunohistochemical results showed that the expression of Clic1 and Atf3 increased in AP (Figure 8E). This exploratory AP-specific gene signature needs further validation and may potentially help to guide our future research directions.

## 4. Discussion

Acute pancreatitis is the leading cause of hospitalizations associated with acute gastrointestinal disease. However, the underlying mechanism of the disease remains unclear. At present, there is no clear effective drug for the treatment of AP. An in-depth study of the underlying mechanisms is critical to understanding the barriers and facilitators for successful treatment. To our knowledge, this study is the first to characterize the immune microenvironment of AP by integrating scRNA-Seq and Bulk RNA-Seq data. We successfully identified 24 clusters of cells, which were further divided into 15 types of cells based on canonical marker genes including macrophages, fibroblasts, acinar cells, endothelials, monocytes, neutrophils, progenitor cells, ductal cells, DC-cells, stellate cells, α-cells, β-cells, T cells, B cells, and granulocytes. This study comprehensively showed the immune microenvironment of AP, explored the interaction network between different cell types, defined AP-specific gene signatures, and provided many new directions for basic research on AP.

This study found that apoptosis, autophagy, ferroptosis, oxidative stress, PI3K-Akt, NF-κB, TNF, and other signaling pathways were abnormally regulated during acute pancreatitis. These results are consistent with many previous reports [22,23,24,25,26]. However, we also found significant enrichment in the AGE-RAGE, P53, Hippo, Ras, Rap1, and HIF-1 signaling pathways. These new findings in acute pancreatitis are reported for the first time. Macrophages, as important immune cells, participate in the biological process of inflammatory diseases through phagocytosis. Studies have found that macrophages dominate the pro-inflammatory phase of AP [27]. M1 macrophages can also further amplify the inflammatory response by expressing pro-inflammatory mediators including cytokines [28]. In our study, it was found that the proportion of macrophages was significantly increased in AP. The number of interactions and interaction weight/strength of the macrophages in AP were significantly higher than those in the controls. A study reported that the anti-inflammatory cells of MRC1+ macrophages may be dysfunctional in a mouse model of neuropathic pain, and that returning these cells to a normal state may help with treatment [19]. However, MRC1+ macrophages have not been reported in AP. In addition, F7, Htr2b, Lyz1, and Gdf3 were significantly overexpressed in different subclusters of macrophages, and their roles in AP still need further study.

The main pathological changes in AP are edema and necrosis of acinar cells. Acinar cells are the most abundant cell type in the pancreas. However, in the samples in this study, the proportion of acinar cells was low. A possible reason is that the acinar cells themselves can produce and secrete trypsin. When the acinar cells are stimulated by external physical or chemical stimulation, the trypsinogen in the cells is abnormally activated, and the acinar cells undergo self-digestion, resulting in the death of a large number of acinar cells. The single cells were captured and subjected to single-cell sequencing using the Chromium 10× genomics platform [29]. Three cell types were identified based on marker genes: Amy2a/b^high^, Sqstm1^high^ and Sox4^low^ and Sqstm1^high^ and Sox4^high^ acinar cells. The Amy2a/b^high^ subcluster was mainly composed of normal acinar cells. Previous studies have found that Sqstm1/p62 is an important regulator of the autophagy process [1,15]. Sox4 was found to suppress the host innate immunity to facilitate pathogen infection [30]. Therefore, it is possible that SOX4 also plays a role in promoting inflammation in AP.

In this study, we used the CellChat package to analyze the cell-to-cell interaction network among the 15 cell types. Then, we calculated the probable communication for the ligand–receptor pairs and identified highly expressed ligand–receptor pairs between macrophages and each cell type in the scRNA-Seq data. We found that TNF signaling such as Tnfsf12-Tnfrsf12a mediates signaling between macrophages and acinar cells. The interaction network between immune cells and acinar cells is more complex in AP. Integrated single-cell and transcriptome data were used to analyze AP-specific gene signatures. The GSE188819, GSE65146, and GSE109227 datasets were downloaded from the GEO database to analyze gene sets significantly associated with AP. Eighteen AP-specific genes were found: Clic1, Sat1, Serpina3n, Atf3, Lcn2, Osmr, Ccl9, Hspb1, Anxa2, Krt8, Cd44, Cd9, Hsp90aa1, Tmsb10, Hmox1, Fxyd5, Plin2, and Pnp. These genes may be key molecules involved in AP disease progression.

## 5. Conclusions

To sum up, our study comprehensively depicted the immune microenvironment of AP, explored the interaction network between different cell types, and defined AP-specific gene signatures, providing many new directions for basic research in AP.

## Data Availability

GSE188819, GSE65146, and GSE109227 datasets from the GEO database (https://www.ncbi.nlm.nih.gov/geo/, accessed on 1 September 2022). The data that support the findings of this study are available from the corresponding author.

## Supplementary Materials

The following supporting information can be downloaded at: https://www.mdpi.com/article/10.3390/biom13010078/s1, RNA-seq data and scRNA-seq data are shown in Supplementary Materials. Figure S1. The top 10 marker genes in 24 cell clusters; Figure S2. GSVA analysis in 15 cell types (signaling pathway); Figure S3. The distribution of acinar cells derived from different cell clusters and different sample origins; Table S1. Markers for identifying cell types; Table S2. Marker genes in 15 cell types.
