# Supplementary material for: Integration of scRNA-Seq and Bulk RNA-Seq Reveals Molecular Characterization of the Immune Microenvironment in Acute Pancreatitis
Source: Biomolecules. 2022 Dec 30;13(1):78. doi: 10.3390/biom13010078 (PMC9855877; doi:10.3390/biom13010078)
Supplement: Supplementary file 1 [file biomolecules-13-00078-s001.zip › Table S1.pdf]

Table S1 Markers for identifying cell types.

Celltype	Markers
Acinar-cell	Prss1, Prss2, Cpb1, Ctrb1, Cpa1
Alpha-cell	Nkx6-1, Irx2, Arx, Pax6, Pdx1, Sst
B-cell	B220, Cd45r, Pax5, Igm, Ebf, Br3, Cd21, Cd22, Cd23
Beta-cell	GLUT2, Ngn3, Pdx1, Mafa, Par6
DC-cell	Cd11c, Cd74, Cd83, Cd86, H2-eb1, Cd80
Endothelial-cell	Pecam1, Cd31, Egfl7, Flt1, Emcn, Esam, Kdr, Tek, Cd34, Cdh5
Fibroblasts	Col3a1, Col5a2, Fn1, Gsn, Lrp1, Thy1
Granulocytes	Cd11b, Fc-epsilonri-alpha, Gr-1, Ly6g, Cd117, Cd43, Cd49b, Cebpe, Cd123
Macropahge	Cd68, Cd206, F4/80, Cd11b, Arg-1, Cd16
Monocytes	Cd11b, Ly6c, Csf1r, Slpi, Ccr2, Gr-1, Ly6c2, Ccl5, S100a4
Neutrophil	Ly6G, Ssc, Cebpe, Csf3r, Gr-1, Wfdc17, Ccl6
Ductal-cell	Epcam, Krt18, Krt19, CK7, Spp1
Progenitor-cell	c-Kit, c-Met, Dclk1
Stellate cells	Des, Gfap, Acta2
T-cell	Cd3, Cd4, Cd45, Cd8, Cd2, Cd25, Cd5, Cd44, Cd127, Cd62l, Foxp3, Cd73, Mel-14, Tcf7, Trbc2, Cd183, Cd194
