# Supplementary figures and images for: Integration of scRNA-Seq and Bulk RNA-Seq Reveals Molecular Characterization of the Immune Microenvironment in Acute Pancreatitis
Source: Biomolecules. 2022 Dec 30;13(1):78. doi: 10.3390/biom13010078 (PMC9855877; doi:10.3390/biom13010078)

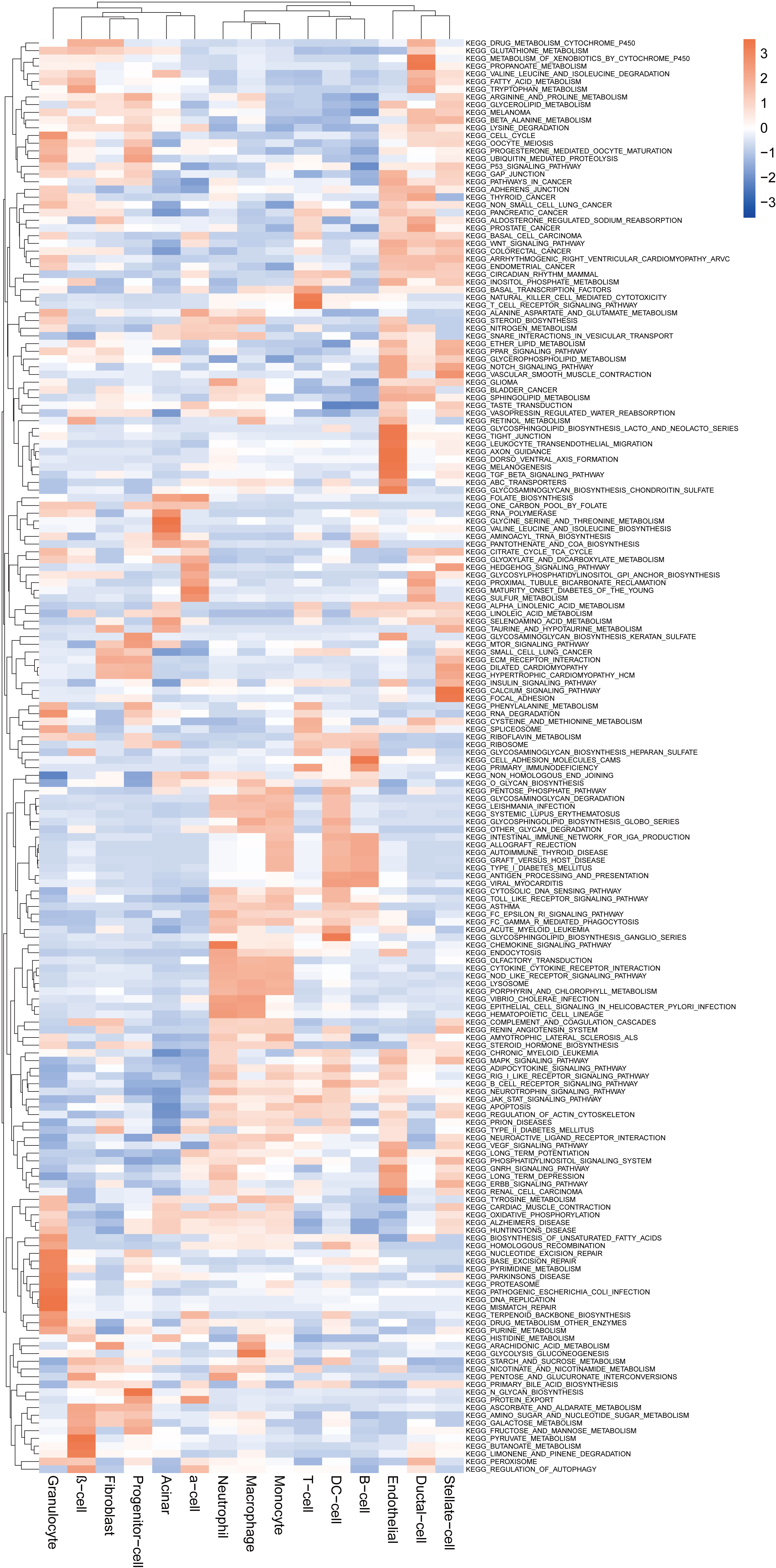

Supplement: Supplementary file 1 [file biomolecules-13-00078-s001.zip › Figure S2.tif]

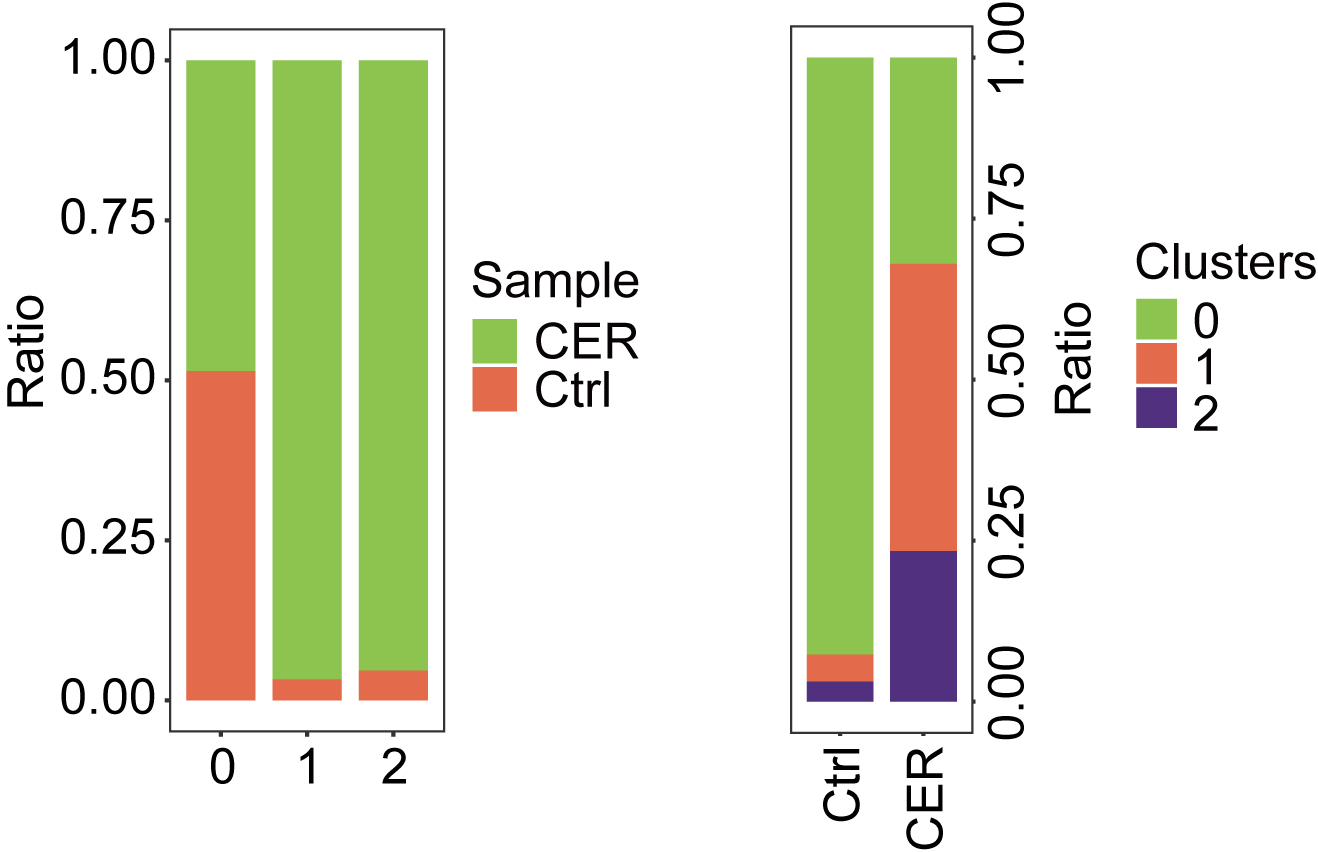

Supplement: Supplementary file 1 [file biomolecules-13-00078-s001.zip › Figure S3.tif]

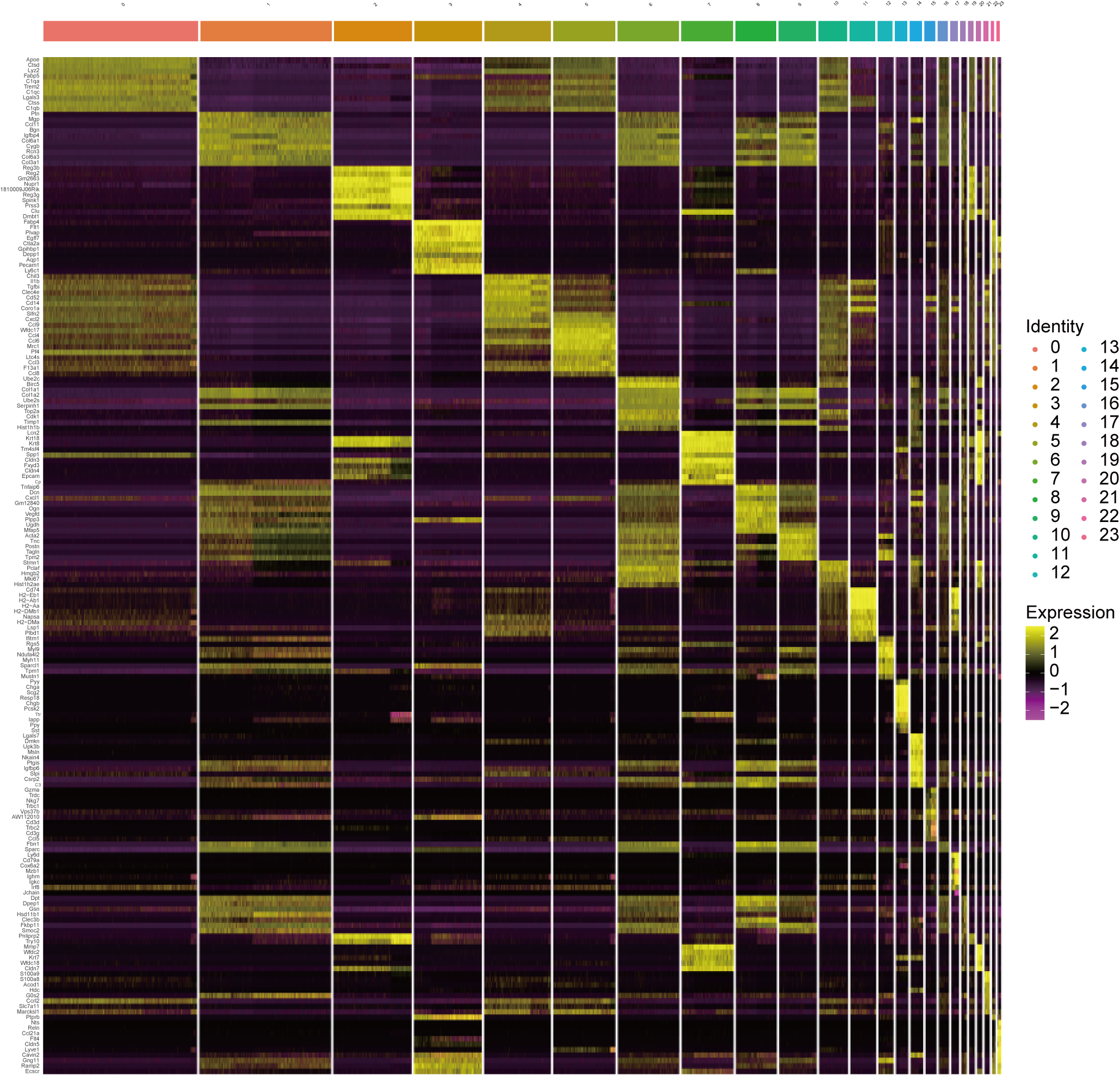

Supplement: Supplementary file 1 [file biomolecules-13-00078-s001.zip › Figure S1.tif]
